# The social and psychological impact of hepatic encephalopathy

**DOI:** 10.1007/s11011-024-01384-x

**Published:** 2024-07-16

**Authors:** Michael Sørensen, Mette Munk Lauridsen, Sara Montagnese

**Affiliations:** 1 Department of Internal Medicine, Viborg Regional Hospital, Viborg, Denmark; 2 Department of Hepatology & Gastroenterology, Aarhus University Hospital, Aarhus, Denmark; 3 Department of Gastroenterology & Hepatology, Institute for Regional Health Research, University Hospital of Southern Denmark, Esbjerg, Denmark; 4 Department of Medicine (DIMED), University of Padova, Padova, Italy; 5 Chronobiology, Faculty of Health and Medical Sciences, University of Surrey, Guildford, UK

## Abstract

Hepatic encephalopathy (HE) is a brain dysfunction caused by liver insufficiency with symptoms ranging from slight cognitive changes detectable only by neuropsychiatric testing to coma. Up to 60% of patients with cirrhosis have mild forms of HE and 35% will at some point experience overt HE. Even in its milder forms, HE impacts the patient’s daily routines, self-sufficiency, quality of life, and, thereby, socio-economic status. HE is a condition affecting the whole household including formal and informal caregivers, who carry a heavy burden. Early identification, prophylaxis, and treatment of HE are essential for relieving patients and informal caregivers.

## Introduction

Hepatic encephalopathy (HE) is a brain dysfunction caused by liver insufficiency or portal-systemic shunt (AASLD and EASL 2014; EASL 2022). It is a common complication of late-stage cirrhosis regardless of aetiology that manifests as a broad spectrum of neurological and psychiatric symptoms ranging from subtle neuropsychiatric alterations (covert HE) to coma. Noteworthy, HE is also identified in up to 60% of patients with non-cirrhotic portal hypertension, porto-sinusoidal vascular disease including shunts, and chronic portal vein thrombosis (Gioia et al. 2021). Patients admitted to the hospital with HE have high readmission rates (Di Pascoli et al. 2017) and the one-year mortality rate for patients with alcohol-related cirrhosis who develop HE is 64%, which is higher than that of those who develop ascites, variceal bleeding, or even the combination of the two (Jepsen et al. 2010). HE as a complication to cirrhosis is also the complication most associated with poor health-related quality-of-life (HRQoL) (Rabiee et al. 2021).

As shown in Table 1, HE is graded according to symptoms from covert, being detectable only by neuropsychological tests, to higher grades that are clinically detectable (overt) and often require hospitalisation (Conn et al. 1977; AASLD and EASL 2014). If diagnosed and treated, HE is, for the most part, reversible, but some patients do not return to their baseline performance between HE episodes, and the condition is thus classified as persistent (AASLD and EASL 2014). Table 1 also shows that the symptoms of HE mainly affect cognitive and physical functions and, as discussed here, episodic and persistent HE thus have significant impact on the daily life and socio-economic status of both patients and next-of-kins acting as informal caregivers.

**Table 1:** Identification and grading of hepatic encephalopathy in liver disease — West Haven Criteria including minimal

ISHEN	Description	Suggested operative criteria	West Heaven Grade
Unimpaired	No encephelapathy at all, no history of HE	Tested and proved to be normal	Unimpaired
Covert HE	Psychometric or neuropsychological alterations of tests exploring psychomotor speed/executive functions or neuropsychological alterations without clinical evidence of mental change	Abnormal results of established psychometric or neuropsychological tests without clinical manifistations	Minimal
	• Trivial lack of awareness • Euphoria or anxiety • Shortened attention span • Impairment of addition or subtraction • Altered sleep rhythm	Despite orientated in time and space (see below), the patient appears to have some cognitive/behavioural decay with respect to his/her standard on clinical examination, or to the caregivers	Grade 1
Overt HE	• Lethargy or apathy • Disorientation for time • Obvious personality change • Inappropriate behaviour • Dyspraxia • Asterixis	Disorientated for time (at least three of the followings are wrong: day of the month, day of the week, month, season or year) ± the other mentioned symptoms	Grade 2
	• Somnolens to semi-stupor • Responsive to stimuli • Confused • Gross disorientation • Bizarre behaviour	Disorientated also for space (at least three of the following wrongly reported: country, state [or region], city or place) ± the other mentioned symptoms	Grade 3
	Coma	Does not respond to even pain stimuli	Grade 4

Adapted from (AASLD and EASL 2014)

### The burden of HE on patients and informal caregivers

Liver disease itself has a significant impact on daily life as patients often experience loneliness, stigmatisation, anxiety as well as physical limitations including fatigue (Gronkjaer and Lauridsen 2021; Carbonneau et al. 2018; Rajpurohit et al. 2024a, b; Rabiee et al. 2021). The neuropsychological alterations associated with HE cause the daily life of a patient to be further disturbed by daytime sleepiness, impaired general daily functioning, and reduced vigilance (Fabrellas et al. 2020; Faccioli et al. 2022; Kunzler-Heule et al. 2016; Montagnese et al. 2012; Montagnese and Bajaj 2019; Louissaint and Tapper 2020). Symptoms such as reduced reflexes and lapses of consciousness lead to impaired driving skills, and current guidelines and expert consensus accordingly recommend that patients avoid driving after a bout of overt HE (Kircheis et al. 2009; EASL 2022). Collectively, cognitive problems and the inability to work cause a high rate of unemployment among patients with previous HE, which, along with health care expenses and several other factors related to cirrhosis, negatively impacts financial status which leads to further social isolation (Bajaj et al. 2011).

Patients are often cared for by next-of-kins, close relatives, friends, or neighbours, who act as informal caregivers (Fabrellas et al. 2020; Louissaint and Tapper 2020; Saleh et al. 2022; Shrestha et al. 2020). For many patients and informal caregivers, the first bout of overt HE is frightening and unexpected because they were not aware of the risk of HE associated with cirrhosis. This unpreparedness and the profound, albeit temporary, loss of autonomy caused by HE can lead caregivers to fear the patient’s imminent death or severe neuropsychiatric disorders such as fast-onset Alzheimer’s disease, stroke, or head trauma (Fabrellas et al. 2020; Kunzler-Heule et al. 2016; Orr et al. 2014). For milder forms of HE, symptoms such as altered speech, slow movements, and clumsiness also cause fear, anxiety, and sorrow among both patients and caregivers (Fabrellas et al. 2020; Montagnese et al. 2012; Saleh et al. 2022). There is a clear association between a patient’s mental status and the psychological burden on caregivers (Montagnese et al. 2012), and the stress introduced by higher job loss and financial insecurity introduces an even heavier caregiver burden (Shrestha et al. 2020; Bajaj et al. 2011; Schomerus and Hamster 2001).

Despite the availability of many tools that can be used to quantify HE symptoms, monitor treatment response, and assess health-related quality of life, these are, unfortunately, rarely used among clinicians (Orr et al. 2014; Lauridsen and Bajaj 2015). Focused liver-specific questionnaires or longer questionnaires such as the Sickness Impact Profile seem to be better for detecting differences in covert HE, whereas more general questionnaires suffice in overt HE (Montagnese and Bajaj 2019) though simple tools actually perform well (Lauridsen et al. 2020). New app-based technologies hold the potential for closer surveillance of daily functioning and quality of life (Shaw et al. 2023).

### Mitigating the impact of HE on daily life

#### Early identification of HE

In general, all patients with chronic liver disease, especially those with active liver disease, are at risk of developing HE. Early identification of patients at risk of developing HE and early diagnosis and treatment of any HE bout have several benefits. Besides affecting the daily lives of patients and informal caregivers, as outlined above, covert HE is also associated with a higher risk of overt HE and, thus, a higher mortality rate, which can be reduced if identified and treated early (Gairing et al. 2024; Parker 2020).

#### Improving health literacy and self-management strategies

HE often catches the patient and caregiver off guard. Further, HE is recurrent in one in three patients, and efforts should be made to provide self-management tools for the patient and caregivers (Table 2). Education of patients and caregivers is essential and should include awareness of the disease, prevention, and treatment (Carbonneau et al. 2018; Mikkelsen et al. 2016; Rodenbaugh et al. 2020; Garrido et al. 2017). Up to 75% of patients with chronic liver disease suffer from muscle wasting, and moderate to severe malnutrition and dietary protein deficiency can cause muscle breakdown and contribute to further muscle wasting as well as ammonia production (Amodio et al. 2013; EASL 2022). It is thus important that patients attempt to maintain or rebuild their muscle mass by exercising and proper diet (Aamann et al. 2019; Tandon et al. 2018; Berzigotti et al. 2016). Patients should avoid longer periods of fasting and a late-night snack is recommended (Leoni et al. 2023). Table 2 summarises some topics that should be addressed when educating patients and caregivers.

**Table 2:** Focus on education of patients and caregivers about hepatic encephalopathy

Awareness	Prevention
Patients and caregivers should be informed about the risk of HE and how to spot the condition. This includes both subtle as well as overt signs of HE. It is important to inform that HE is largely reversible.	Patients and caregivers should be educated about practical tools to help prevent development or recurrence of HE. These include: 1. Early signs of HE – when to contact health care professionals 2. Dietary guidance including protein intake and a late-night snack 3. Rationale for using lactulose and rifaximin including treatment goals 4. Risk and signs of dehydration 5. Signs of infections 6. Abstinence from alcohol and medications such as aspirin, opioids, benzodiazepines, non-steroid anti-inflammatory drugs (NSAIDs)

### Quality of life and patient-reported outcomes as clinical endpoints

Despite the profound effects on the quality of life of both patients and informal caregivers, quality of life and patient-reported outcomes are rarely included as endpoints in clinical studies. A few sporadic studies have included cognition and quality of life as endpoints in studies of hyponatremia (Ahluwalia et al. 2015) and L-carnitine (Yoon et al. 2022). In a recent systematic review of the effects of lactulose and/or rifaximin on patient-reported outcomes, only 16 studies met the inclusion criteria, most studies on covert HE (Moon et al. 2023). It is worth mentioning that both treatments seemed to improve overall health-related quality of life, social activity, sleep, and, to some extent, physical functioning (Moon et al. 2023). As the only recommended pharmaceutical treatments for HE, lactuose has been used for decades and rifaxmin for a little more than a decade. As such, it is staggering that patient-reported outcomes have not been addressed further in clinical trials. We strongly advocate that future studies address these issues using standardized instruments.

In conclusion, more focus on early detection and information to patients, next-of-kin, and caregivers about HE is strongly encouraged as the condition has a tremendous impact on daily life and survival. Quality of life and the burden on informal caregivers should also be addressed in clinical trials of treatments for HE.

## Data Availability

No datasets were generated or analysed during the current study.
